# *Ascosphaera apis* as a target for the antifungal activity of symbiotic Bifidobacteria in honey bees

**DOI:** 10.3389/finsc.2025.1669013

**Published:** 2025-10-01

**Authors:** Massimo Iorizzo, Sonia Ganassi, Bruno Testa, Licia Maria Di Donato, Gianluca Albanese, Mariantonietta Succi, Francesca Coppola, Rosaria Cozzolino, Cristina Matarazzo, Dalila Di Criscio, Cosimo Tedino, Antonio De Cristofaro

**Affiliations:** ^1^ Department of Agricultural, Environmental and Food Sciences, University of Molise, Campobasso, Italy; ^2^ Department of Agricultural Science, University Federico II, Portici, Napoli, Italy; ^3^ Institute of Food Science, National Council of Research (ISA-CNR), Avellino, Italy

**Keywords:** honey bee, gut microbiota, *Bifidobacterium asteroides*, chalkbrood disease, *Ascosphaera apis*

## Abstract

**Introduction:**

The genus *Bifidobacterium* is a key component of the honey bee gut microbiota, playing a fundamental role in maintaining host health and colony well-being. Alongside other core genera such as *Bombilactobacillus*, *Gilliamella*, *Lactobacillus*, and *Snodgrassella*, *Bifidobacterium* contributes to essential functions including nutrient digestion, immune modulation, and protection against pathogens. Among threats to honey bee health, Chalkbrood disease, caused by fungus *Ascosphaera apis*, remains a major concern due to detrimental effects on colony strength and honey yield.

**Materials and methods:**

We characterized enzymatic activity and carbohydrate assimilation of nine *Bifidobacterium* strains isolated from the honey bee intestinal tract. In parallel, we assessed antifungal potential against *A. apis* strains, focusing on volatile organic compounds (VOCs).

**Results and discussion:**

Notably, *Bifidobacterium asteroides* 3CP-2B exhibited enzymatic capabilities supporting digestive functions and metabolism of sugars potentially harmful to honey bees. This strain showed marked antifungal activity against *A. apis*, mediated by volatile and non-volatile bioactive metabolites. Among VOCs identified, propanoic acid, ethanol, acetic acid, ethyl propionate, and 1-propanol were the most prominent compounds associated with the antifungal effect.

## 1 Introduction

In recent years, honey bees (*Apis mellifera* L.) have emerged as an important model system for understanding the functional roles of bacteria within the gut microbiome (1, 2). However, it remains unclear how specific members of the gut microbiota influence bee health and physiological state (3). Honey bee gut is primarily dominated by nine bacterial taxa, which together comprise more than 95% of the total gut microbial community. Among these, five phylogenetic lineages are consistently present in every individual and are defined as the core members of the honey bee gut microbiota. These core lineages represent genus-level taxa from distinct bacterial classes: *Gilliamella* (Gammaproteobacteria), *Snodgrassella* (Betaproteobacteria), *Lactobacillus* Firm-4 (including *Bombilactobacillus*), *Lactobacillus* Firm-5 (including *Apilactobacillus*), and *Bifidobacterium* (Actinobacteria).

This characteristic taxonomic composition of the microbiota, comprising largely species exclusive to social honey bees, along with their essential biochemical contributions to the host, suggests a highly specialized and co-evolved relationship between microbes and honey bees (4, 5). A gut microbiota with a balanced composition plays a crucial role in defending against pathogens and parasites, detoxifying foodborne toxins, and regulating the immunity, metabolism, behavior, and development of honey bees. Conversely, dysbiosis of this community can lead to altered gene expression related to these key functions, potentially compromising overall health and well-being (3).

The genus *Bifidobacterium* encompasses Gram-positive bacteria belonging to the family *Bifidobacteriaceae* within the phylum *Actinomycetota* (6). *Bifidobacterium* spp. are symbiotic microorganisms that contribute to gastrointestinal homeostasis in humans, animals, and insects; in honey bees, they colonize the gut throughout development, with maximal abundance in the adult hindgut (3, 7–10). Although typically less abundant than other core gut taxa, they play a critical role in host metabolism, immune regulation, disease resilience, and adaptation to environmental stressors (11, 12). To date, multiple *Bifidobacterium* species have been identified and characterized from the gut microbiota of various honey bee species within the family *Apidae* (**Table 1**). Recently, *Bifidobacterium favimelis*, a novel species isolated from black comb honey of *A. mellifera*, was identified by Li et al. (23). The presence and divergence of *Bifidobacterium* strains in honey bees is attributed to a long-term coevolutionary process, reflecting their adaptation to various microenvironments within the bee gut and hive, as well as to hive-mediated vertical transmission across generations (24–27). Populations of bifidobacteria in honey bees have been observed to remain relatively stable over time, suggesting that these microorganisms play a consistent and essential role in host physiology (28, 29). Strains of *Bifidobacterium* inhabiting the honey bee gut are of particular interest due to their potential probiotic properties. For example, *B. asteroides* has been shown to stimulate the production of host-derived hormones, such as prostaglandins and juvenile hormone derivatives, which are known to influence honey bee development (30). Comprehensive genomic analyses have revealed that *Bifidobacterium* species harbor a substantial repertoire of genes involved in carbohydrate metabolism, underscoring their functional role in insect physiology (4, 12, 31). Recent studies on pollinator gut microbiota have further elucidated the involvement of bifidobacteria in maintaining immune function, enhancing disease tolerance, and improving resistance to environmental stressors (11). The genus *Bifidobacterium* supports honey bee health through polysaccharide degradation and immune modulation; however, its abundance and overall gut microbiota stability are influenced by factors such as diet, seasonal changes, caste roles, geography, and exposure to xenobiotics like herbicides and antibiotics, which can disrupt microbial balance and lead to dysbiosis, impairing metabolism and vitamin biosynthesis (14, 29, 31–36). Moreover, a disrupted gut microbiota may increase honey bee susceptibility to parasitic infections, including those caused by *Nosema* spp. and *Ascosphaera apis* (3, 36–40).

**Table 1:** *Bifidobacterium* species isolated from the gut microbiota of different bee species.

Bifidobacterium species	Host insect	Reference
*Bifidobacterium asteroides*	Honeybee (*Apis mellifera*, *Apis cerana*)	(13)
*Bifidobacterium coryneforme*	Honeybee (*Apis mellifera*, *Apis cerana*)	(13)
*Bifidobacterium indicum*	Honeybee (*Apis mellifera*, *Apis cerana*)	(13)
*Bifidobacterium kimbladii*	Honeybee (*Apis mellifera*)	(14)
*Bifidobacterium apicola*	Honeybee (*Apis mellifera*)	(15)
*Bifidobacterium apis*	Honeybee (*Apis mellifera*)	(16)
*Bifidobacterium apousia*	Honeybee (*Apis mellifera*)	(12)
*Bifidobacterium choladohabitans*	Honeybee (*Apis mellifera*)	(12)
*Bifidobacterium polysaccharolyticum*	Honeybee (*Apis mellifera*)	(12)
*Bifidobacterium mellis*	Honeybee (*Apis mellifera*)	(17)
*Bifidobacterium mizhiense*	Honeybee (*Apis mellifera*)	(18)
*Bifidobacterium actinocoloniiforme*	Bumble bees (*Bombus lucorum*)	(19)
*Bifidobacterium bohemicum*	Bumble bees (*Bombus lapidarius*)	(19)
*Bifidobacterium bombi*	Bumble bees (*Bombus terrestris*)	(20)
*Bifidobacterium commune*	Bumble bees (*Bombus hypnorum*)	(21)
*Bifidobacterium xylocopae*	Carpenter bees (*Xylocopa violacea*)	(22)
*Bifidobacterium aemilianum*	Carpenter bees (*Xylocopa violacea*)	(22)

Chalkbrood, caused by the fungus *A. apis*, is a widespread fungal disease that primarily affects developing honey bee brood, especially in *A. mellifera* colonies, although it can also impact various other bee taxa (41–44). Recent evidence indicates an increasing global incidence of chalkbrood, which is contributing to honey bee population declines and significant reductions in colony productivity (45–48); moreover, it has been shown that chalkbrood infection alters the honey bee gut bacteriome and increases the host’s vulnerability to other pests and pathogens (49–52). *A. apis* is generally regarded as an opportunistic pathogen that is efficiently dispersed and highly prevalent; however, its mere presence in the hive does not necessarily lead to disease manifestation. Rather, one or more predisposing factors must coincide for a clinical outbreak to occur. These include environmental stressors such as damp and cold weather, colony health status, genetic susceptibility, and developmental stress within the brood (53, 54). Infection is initiated when larvae orally ingest fungal ascospores, which subsequently germinate in the posterior midgut. The resulting hyphae invade the epithelial cells and basement membrane, ultimately leading to larval death. Fungal development continues in a necrotrophic phase even after the host’s demise (55).

Over the years, various chemotherapeutic agents have been investigated for their efficacy against *A. apis* (53, 56), but, none have proven effective in preventing chalkbrood, despite their antifungal activity. Moreover, the presence of antifungal residues in honey represents a potential health hazard for consumers (57). Consequently, there is a growing demand for eco-friendly, and sustainable alternatives for disease control (58–63). In this context, the use of microbial resources as biocontrol agents against honey bee pathogens, including *A. apis*, offers promising opportunities (64–66). Several studies have demonstrated that *Apilactobacillus kunkeei* and *Lactiplantibacillus plantarum*, isolated from the honey bee gut, can inhibit *A. apis* mycelial growth *in vitro*, suggesting their potential as prophylactic agents to restore and maintain gut microbial balance (56, 67). Similarly, other beneficial microbes have shown effectiveness in the biocontrol of chalkbrood (68, 69). Notably, Daisley et al. (70) demonstrated that hive treatments with a probiotic formulation containing *L. plantarum*, *Lacticaseibacillus rhamnosus*, and *A. kunkeei* exerted strong antifungal effects against *A. apis* while also promoting the recovery of symbiotic gut communities. These microbiome shifts were positively correlated with enhanced brood production and colony development (70).

To date, there is limited research on the use of *B. asteroides* as an anti-fungal or probiotic agent in beekeeping (71). In a study by Alberoni et al. (28), symbiotic species including *B. asteroides*, *B. coryneforme*, and *B. indicum* were shown to enhance colony productivity when administered in sugar syrup as a dietary probiotic. Additionally, *Bifidobacterium* spp. supplementation led to reduced *Nosema* infection rates in honey bee colonies (11). More recently, Dengiz et al. (72) reported significant antimicrobial activity by *B. asteroides*, *B. choladohabitans*, and *B. polysaccharolyticum* against key bee pathogens such as *Paenibacillus larvae*, *Melissococcus plutonius*, and *Serratia marcescens*. Conversely, a *Bifidobacterium bifidum* strain, isolated from human feces, did not exhibit inhibitory effect on *A. apis* mycelial growth (73). This finding supports the growing consensus that exogenous probiotics, not derived from the honey bee microbiota, may lack beneficial effects, or even pose risks, to health (3, 74–77). Therefore, the identification and characterization of autochthonous *Bifidobacterium* strains with honey bee-specific probiotic properties is essential for developing effective, safe, and sustainable tools for disease prevention in apiculture.

In the present study, a preliminary characterization of *Bifidobacterium* strains isolated from the gastrointestinal tract of *A. mellifera* (collected from apiaries in central-southern Italy) was conducted, including analyses of enzymatic activity and carbohydrate assimilation profiles. Furthermore, the antifungal activity of these strains against multiple *A. apis* isolates was evaluated, with particular focus on the production of volatile organic compounds (VOCs). This is the first report describing the potential of *B. asteroides* as a biocontrol agent against chalkbrood disease.

## 2 Materials and methods

### 2.1 Fungal cultures

**Table 2** provides a detailed overview of the *A. apis* strains employed in the present study.

**Table 2:** Catalogue of *A. apis* strains utilized in this study, accompanied by their GenBank accession numbers from the National Center for Biotechnology Information (NCBI).

Fungal strain ID	Taxonomical identification	Accession number
1A1R 2.2	*Ascosphaera apis*	PV056024
1B3R (1)	*Ascosphaera apis*	PV056025
1A1R 1.1	*Ascosphaera apis*	PV056026
1A3R (2)	*Ascosphaera apis*	PV056027
1B1R (1)	*Ascosphaera apis*	PV056028
1A3R 1.1	*Ascosphaera apis*	PV056029
CB2	*Ascosphaera apis*	PV056030
CB3	*Ascosphaera apis*	PV056031
1A1R 1.2	*Ascosphaera apis*	PV056032
AA	*Ascosphaera apis*	PV056033
CB1	*Ascosphaera apis*	PV056034
1A2R 1.2	*Ascosphaera apis*	PV056035
1B2R 2.2	*Ascosphaera apis*	PV056036
1B2R 2.1	*Ascosphaera apis*	PV056037
CB4	*Ascosphaera apis*	PV056038

### 2.2 Isolation of bifidobacteria

Worker bees (*A. mellifera* subsp. *mellifera*) were collected from managed apiaries in the Molise and Campania regions (central-southern Italy). After euthanization by rapid cooling on ice, bees were transported under refrigeration to the laboratory on the same day and stored at −80 °C until DNA extraction. Dissection of the intestinal tract was performed under sterile conditions using stainless-steel scissors. Entire guts were placed in sterile glass Petri dishes with physiological saline solution (0.9% NaCl) and homogenized. Serial dilutions of the homogenates were plated on Bifidobacterium Selective Medium agar (BSM; Sigma-Aldrich) and incubated at 37 °C for 72 h under anaerobic conditions (Anaerogen system, Oxoid, Milan, Italy). Colonies showing Gram-positive staining with characteristic bifurcated (Y- or V-shaped), club-shaped, or spatula morphologies were presumptively identified as *Bifidobacterium*.

### 2.3 Genotypic characterization

Genomic DNA (gDNA) was extracted using the Bacterial Genomic DNA Isolation Kit (Norgen Biotek, Thorold, ON, Canada) according to the manufacturer’s instructions. The 16S rRNA gene was amplified by PCR using universal primers 27F (5′-AGAGTTTGATCCTGGCTCAG-3′) and 1492R (5′-TACGGTTACCTTGTTACGACTT-3′). Each 20 µL reaction included 1× Master Mix (Norgen Biotek), 2.5 µM of each primer, and 10 ng of template DNA. Negative controls using Milli-Q water were included. PCR was performed using a Mastercycler Nexus (Eppendorf, Hamburg, Germany) with cycling parameters: 95 °C for 5 min; 35 cycles of 95 °C for 30 s, primer-specific annealing temperature for 1 min, 72 °C for 1.5 min; followed by a final extension at 72 °C for 5 min. Products were resolved on a 1% (*w*/*v*) agarose gel in 1× TAE buffer, visualized under UV light (Bio-Rad, Hercules, CA, USA), and compared against a 1 kb DNA ladder (Norgen Biotek). Amplicons were purified using the QIAquick PCR Purification Kit (QIAGEN GmbH, Hilden, Germany) and sequenced (Eurofins MWG Biotech, Ebersberg, Germany). Sequences were analyzed using BLAST (78) against the NCBI nucleotide database (NCBI 79). Strains with ≥98% identity were assigned species-level taxonomy (80).

### 2.4 Biochemical characterization

#### 2.4.1 Carbohydrate assimilation patterns

Carbohydrate utilization was tested using Fermentation Broth Base (FBB; Biolife, Milan, Italy) supplemented with bromocresol purple as a pH indicator. Prior to testing, bacterial strains were cultured in BSM broth at 37 °C for 48 hours under anaerobic conditions. Cultures were centrifuged, and the cell pellet washed with 0.9% NaCl solution to remove the residual medium. The pellet was then resuspended in saline to reach a standard turbidity of 0.5 McFarland (approx. 1.5 × 10^8^ CFU/mL) (81), and used as inoculum. Thirteen carbohydrates were tested: D-arabinose, fructose, galactose, glucose, lactose, maltose, mannose, melezitose, melibiose, raffinose, rhamnose, sucrose, and xylose. For each assay, 4.5 mL of FBB were mixed with 500 μL of carbohydrate solution and 100 μL of bacterial suspension. Negative controls were prepared identically but without inoculum. Sugar solutions were sterilized using 0.22 μm syringe filters. Assays were incubated at 37 °C for 48 hours under anaerobic conditions. The change of color from purple to yellow is due to the production of acids during fermentation. All tests were performed in triplicate for each strain–carbohydrate combination.

#### 2.4.2 Enzymatic profile

Enzymatic activity was evaluated using the API ZYM system (BioMérieux, Lyon, France). The cell pellet (CP), prepared as above, was resuspended in 0.9% NaCl solution to achieve a turbidity of 5 McFarland. Wells of the API ZYM strip were inoculated with 65 µL of this suspension and incubated at 37 °C. After 4 hours, enzymatic activity was assessed based on color change, according to the manufacturer’s guidelines.

#### 2.4.3 Biogenic amine production

Biogenic amine production by *Bifidobacterium* strains was qualitatively assessed using method from Torracca et al. (82), with slight modifications. The *Bifidobacterium* strains were grown in BSM broth at 37 °C for 48 h anaerobically. Subsequently, the cultures, in a volume of 50 µL, were inoculated using a spot inoculation method onto solid media formulated with the following components: 0.5% tryp-tone, 0.5% yeast extract, 0.5% meat extract, 0.25% NaCl, 0.05% glucose, 0.1% Tween 80, 0.02% MgSO_4_, 0.005% MnSO_4_, 0.004% FeSO_4_, 0.2% ammonium citrate, 0.001% thia-mine, 0.2% K_2_HPO_4_, 0.01% CaCO_3_, 0.005% pyridoxal-5-phosphate, and 1.5% agar. The medium was supplemented with 1% of each amino acid precursor: L-histidine, L-tyrosine, L-lysine monohydrate, and L-ornithine monohydrochloride. Bromocresol purple (0.006%) was incorporated as a pH indicator, and the medium pH was adjusted to 5.3 prior to inoculation. Petri dishes were incubated at 37 °C for 72 hours under anaerobic conditions. The decarboxylation of the amino acids to the corresponding biogenic amines results in an increase in pH, detected by the culture medium color change. A purple coloration indicated the production of histamine, cadaverine, or putrescine, while medium de-colorization suggested tyramine production. Negative controls lacking amino acid precursors were included to confirm the specificity of the reactions. All tests were conducted in triplicate and all reagents were purchased from Merck KGaA (Darmstadt, Germany).

### 2.5 Antifungal activity assessment

#### 2.5.1 Preliminary evaluation of antifungal activity

Antifungal activity of *Bifidobacterium* strains was assessed using a method adapted from Iorizzo et al. (69). Three matrices were tested: broth culture (BC), cell-free supernatant (CFS), and CP. Bifidobacteria were grown in BSM broth at 37 °C for 48 hours under anaerobic conditions to a final cell density of 10^8^ CFU/mL. BC was collected without further treatment. For CFS, 5 mL of bacterial culture was centrifuged at 8000 rpm for 15 min at 4 °C, and the supernatant filtered through a 0.22 μm cellulose acetate membrane. CP was prepared by washing and resuspending the pellet in 5 mL of sterile distilled water. Antifungal assays were conducted by transferring a 6 mm mycelial disc of *A. apis*, pre-cultured on Sabouraud Dextrose Agar (SDA) at 30 °C for 3 days, to the center of 90 mm Petri dishes containing fresh SDA. In different plates containing the pathogenic fungus, 5 mL of each matrix (BC, CFS, or CP) was added alternately. An SDA plate containing only the pathogenic fungus was used as a control. All plates were incubated aerobically at 30 °C, and each experimental condition was tested in triplicate. Following six days of incubation, the radial growth of *A. apis* mycelium was measured using a digital caliper. The percentage of mycelium radial growth inhibition (% I) was calculated according to the formula: % I = [(C – T)/C] × 100 (83), where *C* represent the radial growth in the control, and *T* represents the radial growth in the presence of different matrices obtained from the *Bifidobacterium* cultures.

#### 2.5.2 Antifungal activity of the VOCs produced by *Bifidobacterium*

The antifungal activity of VOCs produced by *Bifidobacterium* strains was evaluated using a modified double-dish system (DDS) based on Ruiz-Moyano et al. (84). A 100 μL aliquot of a 48-hour culture (10^8^ CFU/mL) was spread on BSM agar in 90 mm Petri dishes. Simultaneously, a 6 mm disc of *A. apis* mycelium (grown on SDA) was placed in the center of a separate plate. Lids of both plates were removed and the dishes sealed together in an inverted DDS configuration using Parafilm (Pechiney Plastic Packaging Co., Milwaukee, WI, USA), with the fungal plate on the bottom. This setup allowed VOCs to diffuse freely. Control DDS setups (no bacteria) were included. After 6 days at 30 °C, radial growth inhibition was measured as previously described. All experiments were performed in triplicate.

### 2.6 Volatile organic compounds profiling

#### 2.6.1 VOCs extraction

**Figure 1:**
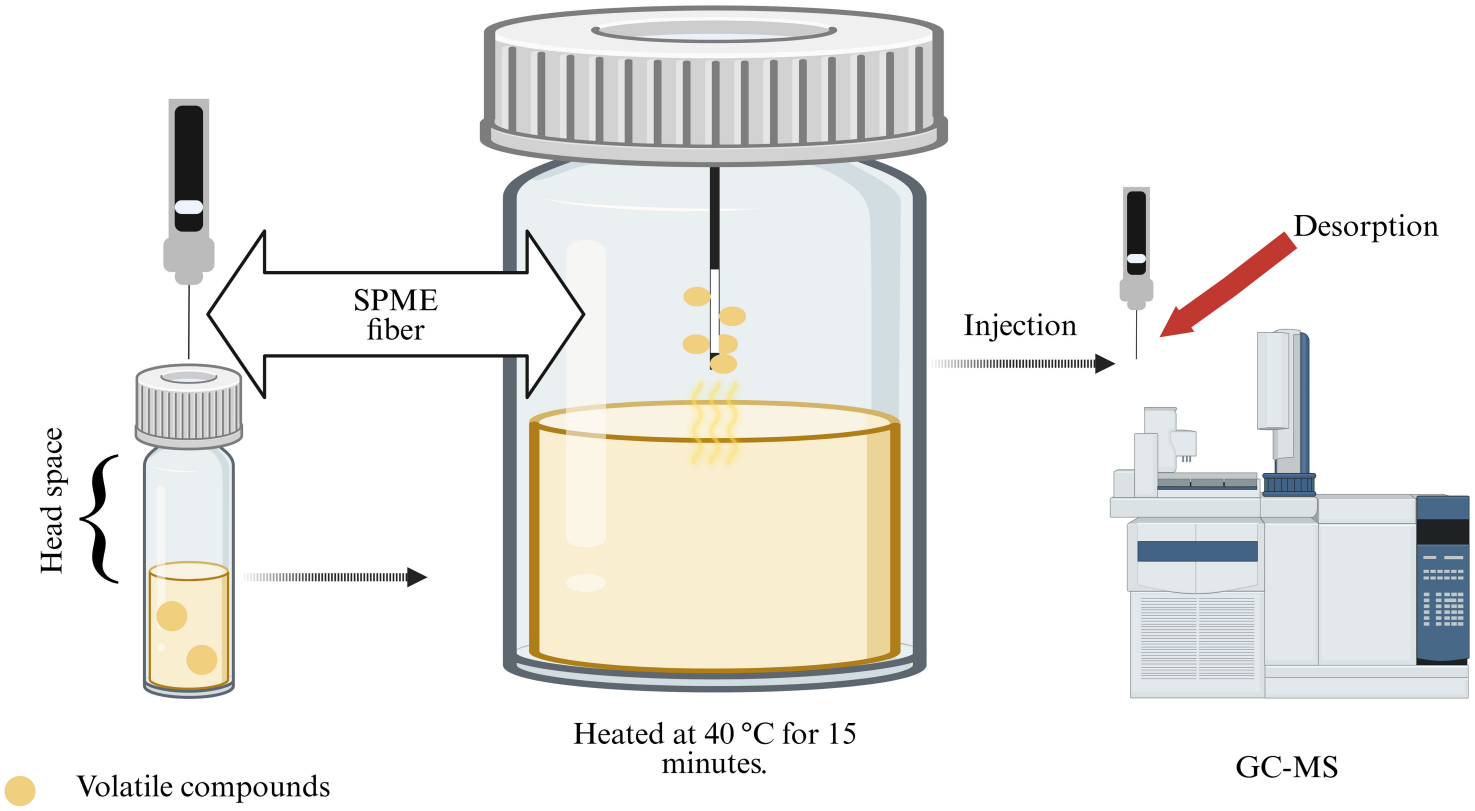
Experimental workflow used to evaluate the production of volatile organic compounds (VOCs) by *B asteroides* 3CP-2B. VOCs were subsequently captured using a solid-phase microextraction (SPME) fiber and were analyzed using gas chromatography coupled with mass spectrometry (GC-MS) (Created with BioRender.com).

VOCs were extracted using headspace solid-phase microextraction (HS-SPME) (**Figure 1**). *B. asteroids* 3CP-2B was cultured for 72 h at 30°C in BSM medium (15 mL) directly in 30 mL screw capped SPME vials (Agilent Technologies, Santa Clara, CA, USA). The vials were sealed with a magnetic screw capped PTFE/silicone liner septum. After 72 hours, the vial was equilibrated to 40 °C for 15 minutes to allow equilibration of the headspace. A 2 cm DVB/CAR/PDMS fiber (50/30 µm; Supelco, Bellefonte, PA, USA) was then inserted into the vial headspace and exposed at 40 °C for 30 minutes to adsorb volatile compounds. Following adsorption, the fiber was immediately transferred to the GC injector port, where desorption was performed at 240 °C for 10 minutes in splitless mode. To distinguish bacterial VOCs from background volatiles, control vials containing non-inoculated media were processed in parallel. Blank runs were also conducted between samples to confirm the absence of carryover or contamination throughout the extraction and analytical procedures. The experiment was done in triplicate.

#### 2.6.2 Gas chromatography–mass spectrometry analysis

Analysis of volatile organic compounds was conducted using a GC–MS system consisting of an Agilent 7890A gas chromatograph coupled with a 5975A mass selective detector (Agilent Technologies, Santa Clara, CA, USA). Chromatographic separation was achieved using a polar HP-Innowax capillary column (30 m × 0.25 mm i.d., 0.50 μm film thickness; Agilent Technologies). The method was adapted from Serradilla et al. (85), with the oven temperature program set as follows: initial hold at 40 °C for 3 minutes; ramp to 150 °C at 4 °C/min, held for 1 minute; then increased to 220 °C at 3 °C/min with a final hold for 2 minutes. Helium was employed as the carrier gas at a constant flow rate of 1.0 mL/min. The injector was operated in splitless mode, and the desorbed VOCs were introduced directly into the ion source. Electron impact (EI) ionization was performed at 70 eV. The ion source and quadrupole temperatures were set to 230 °C and 150 °C, respectively. Mass spectra were acquired in full-scan mode over a range of *m/z* 30–300. Compound identification was performed by comparing the obtained mass spectra and retention indices (linear retention indices, LRI) with entries in the NIST05 and Wiley07 spectral libraries. When available, identification was further confirmed using authentic standards. Semi-quantitative analysis of each VOC was expressed as the relative peak area (RPA%), calculated as the ratio of the individual compound’s area to the total area of all detected VOCs in the total ion chromatogram (TIC).

### 2.7 Statistical analysis

Statistical analyses were conducted in Rstudio (R version 4.3.0). Results from triplicate experiments were expressed as mean ± standard deviation (SD). One-way ANOVA followed by Tukey’s *post hoc* test was used to determine significant differences (*p* < 0.05).

## 3 Results

### 3.1 Taxonomical identification

The nine isolates have been identified as members of the species *B. asteroides, B. apousia, B. mizhiense* and *B. choladohabitans* as reported in **Table 3**.

**Table 3:** List of *Bifidobacterium* strains isolated in this study with their taxonomic assignment and NCBI GenBank accession number.

Bacterial strain ID	Taxonomical identification	% identity	Accession number
1CP-1B-B	*B. apousia*	99.67	PV053127
3CP-6B	*B. apousia*	99.04	PV053134
3CP-10B1	*B. asteroides*	99.47	PV053124
3CP-2B	*B. asteroides*	99.73	PV053131
3CP-8BG1	*B. asteroides*	99.38	PV053133
3CP-3B	*B. asteroides*	98.99	PV053126
3CP-1G	*B. choladohabitans*	99.41	PV053144
1CP-3BG	*B. mizhiense*	99.83	PV053125
1CP-10B	*B. mizhiense*	99.43	PV053128

### 3.2 Biochemical characterization

The enzymatic activities of the bacterial isolates were comprehensively assessed using the qualitative API ZYM kit, with results summarized in **Table 4**. The enzymatic profiles of the *Bifidobacterium* isolates, including *B. asteroides*, *B. apousia*, *B. mizhiense*, and *B. choladohabitans*, revealed activities for several enzymes, notably leucine arylamidase, naphthol-AS-BI-phosphohydrolase, β-galactosidase, and β-glucosidase. Enzymatic activities varied within the *B. asteroides* species. For example, *B. asteroides* 3CP-3B exhibited α-galactosidase activity, which was absent in other strains of the same species. Additionally, strains 3CP-10B1S and 3CP-8BG1B were the only ones to display α-glucosidase activity. Strains 3CP-10B1, 3CP-3B, and 3CP-8BG1B also showed α-mannosidase activity, while strain 3CP-2B was unique in exhibiting α-fucosidase activity. Among *B. apousia* strains, 1CP-1B-B was distinct in showing both esterase and α-glucosidase activities. In contrast, *B. apousia* 3CP-6B-B was the only strain within its species to demonstrate α-mannosidase activity. No enzymatic differences were observed between the two *B. mizhiense* strains.

**Table 4:** Enzymatic profiles of the nine *Bifidobacterium* strains assessed using the API ZYM system.

Enzyme	*Bifidobacterium asteroides* strains				*Bifidobacterium apousia* strains		*Bifidobacterium mizhiense* strains		*Bifidobacterium choladohabitans*
	3CP-10B1S	3CP-3B	3CP-2B	3CP-8BG1B	3CP-6B-B	1CP-1B-B	1CP-3BGS	1CP-10B	3CP-1G
Alkaline phosphatase	–	–	–	–	–	–	–	–	–
Esterase (C4)	–	–	–	–	–	+	–	–	+
Esterase lipase (C8)	–	–	–	–	–	–	–	–	–
Lipase (C14)	–	–	–	–	–	–	–	–	–
Leucine arylamidase	+	+	+	+	+	+	+	+	+
Valine arylamidase	–	–	–	–	–	–	–	–	–
Cystine amyralidase	–	–	–	–	–	–	–	–	+
Trypsin	–	–	–	–	–	–	–	–	–
α-chymotrypsin	–	–	–	–	–	–	–	–	–
Acid phosphatase	+	+	+	+	+	+	+	+	–
Naphthol-AS-BI-phosphohydrolase	+	+	+	+	+	+	+	+	+
α-galactosidase	–	+	–	–	+	+	+	+	+
β-galactosidase	+	+	+	+	+	+	+	+	+
β-glucuronidase	–	–	–	–	–	–	–	–	–
α-glucosidase	+	–	–	+	–	+	–	–	+
β-glucosidase	+	+	+	+	+	+	+	+	+
N-acetil-β-glucosaminidase	–	–	–	–	–	–	–	–	–
α-mannosidase	+	+	–	+	+	–	+	+	+
α-fucosidase	–	–	+	–	–	–	+	+	+

### 3.3 Carbohydrate assimilation profiles and biogenic amines production

The carbohydrate assimilation abilities of the nine *Bifidobacterium* strains are detailed in **Table 5**. All strains were capable of metabolizing fructose, glucose, maltose, melibiose, raffinose, and sucrose. In contrast, none of the strains metabolized D-arabinose or rhamnose. Assimilation of lactose was strain-specific and limited to *B. apousia* 1CP-1B-B, *B. mizhiense* 1CP-10B, and *B. choladohabitans* 3CP-1G. Regarding the production of biogenic amines, none of the strains were able to synthesize these compounds from the tested amino acid precursors.

**Table 5:** Carbohydrate assimilation profiles of the nine *Bifidobacterium* strains.

Carbohydrate	*Bifidobacterium asteroides*				*Bifidobacteria apousia*		*Bifidobacteria mizhiense*		*Bifidobacterium choladohabitans*
	3CP-10B1S	3CP-3B	3CP-2B	3CP-1G	3CP-6B-B	1CP-1B-B	1CP-3BGS	1CP-10B	3CP-1G
D-Arabinose	-	-	-	-	-	-	-	-	–
Fructose	+	+	+	+	+	+	+	+	+
Galactose	+	+	+	+	+	+	+	+	+
Glucose	+	+	+	+	+	+	+	+	+
Lactose	-	-	-	-	-	+	-	+	+
Maltose	+	+	+	+	+	+	+	+	+
Mannose	+	+	+	+	+	-	+	-	+
Melezitose	+	+	+	+	+	+	+	+	–
Melibiose	+	+	+	+	+	+	+	+	+
Raffinose	+	+	+	+	+	+	+	+	+
Rhamnose	-	-	-	-	-	-	-	-	–
Sucrose	+	+	+	+	+	+	+	+	+
Xylose	+	+	+	+	+	+	+	+	+

(+ positive; – negative).

### 3.4 Screening of antifungal activity by *Bifidobacterium* strains

**Table 6** presents the antifungal activity of the nine *Bifidobacterium* isolates against *A. apis* CB3. In most cases, the use of unprocessed culture broth BC resulted in complete inhibition (100%) of fungal growth. Notable exceptions were isolates 3CP-8BG1B and 3CP-6B-B, which exhibited inhibition rates of 76.7% and 98.9%, respectively. The CFS also showed strong antifungal activity, with inhibition percentages ranging from 65.9% (*B. asteroides* 3CP-8BG1B) to 100% (*B. asteroides* 3CP-2B). Regarding the CP fraction, *B. mizhiense* 1CP-10B exhibited the lowest inhibition (35%), whereas strains 3CP-2B and 1CP-3BGS achieved full inhibition (100%). In terms of VOCs, overall inhibition levels were modest. However, strains 3CP-2B and 3CP-8BG1B showed moderate activity, with inhibition values of 54.2% and 47.2%, respectively.

**Table 6:** Inhibitory effects (%) of *Bifidobacterium* strains against *A. apis* CB3 using different matrices: broth culture (BC), cell-free supernatant (CFS), cell pellet (CP), and volatile organic compounds (VOCs).

*Bifidobacterium* strains									
Matrices	3CP-10B1S	3CP-3B	3CP-2B	3CP-8BG1B	3CP-6B-B	1CP-1B-B	1CP-3BGS	1CP-10B	3CP-1G
BC	100.0 ± 0.0^a^	100.0 ± 0.0^a^	100.0 ± 0.0^a^	76.7 ± 0.1^c^	98.9 ± 0.1^b^	100.0 ± 0.0^a^	100.0 ± 0.0^a^	100.0 ± 0.0^a^	100.0 ± 0.0^a^
CFS	75.0 ± 0.6^c^	77.2 ± 0.6^b^	100.0 ± 0.0^a^	65.9 ± 0.7^e^	71.7 ± 0.6^d^	75.3 ± 0.7^c^	70.9 ± 0.9^d^	67.5 ± 0.3^e^	67.3 ± 0.5^e^
CP	52.8 ± 0.6^c^	47.5 ± 0.5^e^	100.0 ± 0.0^a^	54.7 ± 0.8^b^	46.1 ± 0.5^e^	49.2 ± 0.4^d^	100.0 ± 0.0^a^	35.0 ± 0.6^f^	49.1 ± 0.3^d^
VOCs	11.9 ± 0.7^f^	38.2 ± 0.6^c^	54.2 ± 0.3^a^	47.2 ± 0.6^b^	6.1 ± 0.5^h^	24.9 ± 0.4^e^	8.3 ± 0.5^g^	33.9 ± 0.6^d^	6.1 ± 0.5^h^

Subsequently, *B. asteroides* 3CP-2B, the most effective strain, was selected as the reference bacterium for further antifungal assays against all *A. apis* strains listed in **Table 2**.

**Figure 2:**
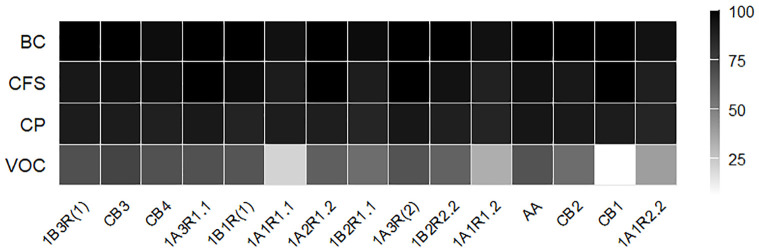
Heatmap showing the inhibition percentages of *A apis* strains by *B asteroides* 3CP-2B across different matrices (BC, CFS, CP and VOC).

The inhibitory activity of *B. asteroides* 3CP-2B against *A. apis* strains was assessed under four different treatments: BC, CFS, CP, and VOCs (**Figure 2**). Overall, the BC treatment exhibited the highest and most consistent antifungal efficacy, with most strains achieving complete inhibition. The CFS treatment also resulted in high inhibition levels, ranging from 88.0% (1A1R 1.2) to 100.0% (1A3R 1.1, 1A2R 1.2, 1A3R (2) and CB1). The CP treatment led to moderately reduced inhibition (from 85.6% to 93.3%). VOCs were the least effective treatment, with inhibition ranging widely from 6.2% (CB1) to 72.8% (CB3), and statistically significant differences among nearly all strains.

### 3.5 Profiling of volatile organic compounds

**Supplementary Table 1** lists all 37 VOCs detected by GC–MS analysis, along with their semi-quantitative relative peak area (RPA%) data. Based on peak areas, the major compounds detected were propanoic acid (45.8%), ethanol (25.0%), acetic acid (17.3%), ethyl propionate (3.1%), 1-propanol (2.3%), isoamyl alcohol (1.7%), propyl propionate (0.7%), ethyl acetate (0.4%), butanoic acid (0.3%), benzaldehyde (0.2%), and 2-methyl-propanoic acid (0.2%) (**Table 7**).

**Table 7:** Main Volatile Organic Compounds (VOCs) produced by *Bifidobacterium asteroides* 3CP-2B detected by HS-SPME/GC-MS with corresponding %RPA.

Volatile compounds	Code	%RPA	^A^KIsp/KIt	^B^ID
Aldehydes				
Benzaldehyde	Ald2	0.2	1518/1520	RI/MS/S
Esters				
Ethyl acetate	E1	0.4	860/863	RI/MS/S
Ethyl propionate	E2	3.1	942/946	RI/MS/S
Propyl propionate	E3	0.7	1045/1047	RI/MS
Alcohols				
Ethanol	Alc1	25.0	933/934	RI/MS/S
1-Propanol	Alc3	2.3	1033/1037	RI/MS/S
Isoamyl alcohol	Alc6	1.7	1212/1215	RI/MS/S
Acids				
Acetic acid	A1	17.3	1448/1445	RI/MS/S
Propanoic acid	A2	45.8	1530/1534	RI/MS
2-Methyl-propanoic acid	A3	0.2	1581/1581	RI/MS/S
Butanoic acid	A4	0.3	1632/1630	RI/MS/S

Mean values of 3 samples are calculated as RPA (%). ^a^RIsp: Relative retention indices calculated against n-alkanes (C_8_–C_20_) on HP-Innowax column; RIt: Relative retention indices on polar column reported in literature ^b^Identification method as indicated by the following: RI: Kovats retention index on a on HP-Innowax column; MS: NIST and Wiley libraries spectra; S: co-injection with authentic standard compounds, where commercially available, on the HP-Innowax column. For each metabolite the coefficient of variability of determinations, evaluated as relative standard deviation, was in all cases <10%.

## 4 Discussion

The isolated microbial cultures were identified as members of the species *B. asteroides*, *B. apousia*, *B. mizhiense*, and *B. choladohabitans*.

Among these, *B. asteroides* is of particular interest due to its previously reported oxygen tolerance and its role in carbohydrate metabolism (86). This species metabolizes dietary sugars, including glucose and fructose, and utilizes the malolactic fermentation pathway to convert malic acid into lactate, thereby contributing to the host’s energy metabolism (86). However, functional data on *B. apousia* and *B. mizhiense* remain still scarce. Based on the known metabolic capabilities of other *Bifidobacterium* species, these isolates are hypothesized to play a significant role in sugar degradation, with *B. apousia* potentially involved in hemicellulose breakdown (12). The balance of the honey bee gut microbiota is crucial for host health, with microbial enzymatic activity directly supporting digestive function (87, 88). Notably, the *B. apousia* strain 1CP-1B-B and *B. choladohabitans* 3CP-1G showed esterase activity; a function involved in lipid digestion, and playing a role in detoxification by hydrolyzing or degrading various compounds, including drugs, pesticides, and other xenobiotics (89–92). Honey bees employ a multifaceted detoxification strategy, including enzymatic processes such as those involving cytochrome P450 monooxygenases, glutathione S-transferases, and carboxylesterases. These enzymatic defenses are complemented by behaviors forming a “social detoxification system,” which includes forager discrimination, dilution through pollen mixing, and colony-level food processing via microbial fermentation, reducing the intake of harmful chemicals (93–96, 46). Given the widespread use of insecticides in agriculture, supplementing the honey bee diet with appropriate probiotics, capable of degrading such compounds, may benefit bee health (64, 97, 98).

All tested isolates exhibited leucine arylamidase activity, suggesting a common ability to participate in protein hydrolysis, consistent with other *Bifidobacterium* species (99). This enzymatic function complements the proteolytic capabilities of other core honey bee gut microbes, such as *Snodgrassella alvi* and *Gilliamella apis* (31, 100). Positive activities were also recorded for acid phosphatase, naphthol-AS-BI-phosphohydrolase, β-galactosidase, and β-glucosidase. Among these, β-glucosidase is particularly important for degrading plant-derived polysaccharides such as cellulose and hemicellulose (12, 101), while β-galactosidase catalyzes the hydrolysis of β-D-galactosides, contributing to the digestion of galactose-containing nectar compounds (31). α-Glucosidase activity was detected in *B. asteroides* 3CP-10B1S and 3CP-8BG1B, *B. apousia* 1CP-1B-B, and *B. choladohabitans* 3CP-1G. This enzyme, also secreted by the hypopharyngeal glands of honey bees (52, 102), plays a critical role in maltose hydrolysis and starch degradation (31, 103, 104), thus contributing to the conversion of nectar into honey.

Additional enzymatic functions, including α-galactosidase, α-mannosidase, and α-fucosidase were detected in certain isolates. Although less studied in honey bee-associated *Bifidobacterium*, these enzymes are involved in degrading complex plant oligosaccharides and polysaccharides. They may contribute to digestion in insects and produce prebiotic compounds that support immune modulation in mammals, including humans (105–107). For example, α-galactosidase breaks down complex carbohydrates such as raffinose and stachyose, important for nutrient absorption in insects (108). α-Fucosidase releases terminal fucose residues, which are key to cell–cell communication and host–microbe interactions in mammals (109, 110). α-Mannosidase hydrolyzes mannose-containing carbohydrates and helps produce prebiotic mannooligosaccharides, which promote the growth of beneficial gut bacteria (111).

Previous studies have suggested that the honey bee gut microbiome may facilitate the metabolism of toxic sugars (112–114). In our study, carbohydrate assimilation profiles revealed both intra- and inter-species variability. All isolates effectively utilized a range of mono- and oligosaccharides commonly found in the honey bee gut, including fructose, glucose, maltose, melezitose, melibiose, raffinose, and sucrose. These results align with earlier findings showing that *Bifidobacterium* species are well adapted to the bees’ carbohydrate-rich diet (31, 86). Some sugars present in the honey bee diet, such as galactose, mannose, lactose, raffinose, and xylose, can be toxic due to the absence of necessary host enzymes for their degradation (31, 114–116). Gut symbionts enhance the honey bee’s ability to process complex polysaccharides and detoxify harmful sugars, improving dietary efficiency and resistance to diseases (101, 117).

Regarding antifungal activity, our results demonstrated that the different matrices (BC, CFS, CP, and VOCs) derived from the evaluated *Bifidobacterium* strains were effective (**Table 6**). Variability in antifungal activity likely reflects differences in the types and quantities of antifungal metabolites produced (118). These metabolites, such as lactic acid, acetic acid, phenyl lactic acid (PLA), short-chain fatty acids (SCFAs), proteins, and others, can disrupt fungal cell membranes, causing damage and inhibiting growth (119; 72, 120–122). The antifungal effects of VOCs are mainly attributed to cell wall and membrane disruption, leakage of intracellular contents, and the induction of oxidative stress (123). Bifidobacteria degrade hexose sugars via the “bifid shunt” pathway, in which fructose-6-phosphoketolase (EC 4.1.2.2) plays a key role and serves as a taxonomic marker for the *Bifidobacteriaceae* family. This pathway typically yields 3 moles of acetate and 2 moles of lactate per 2 moles of glucose, though other byproducts, such as ethanol, can also be produced (124). Ethanol and acetic acid are known for their antimicrobial properties, including antifungal activity, through mechanisms such as membrane disruption, protein denaturation, and interference with fungal DNA and protein synthesis (125, 126). Similarly, 1-propanol and other alcohols (e.g., isoamyl alcohol, 1-butanol) exhibit antifungal effects likely through membrane disruption, inhibition of spore germination, and interference with transcription and translation processes (127, 128).

In our study, *B. asteroides* 3CP-2B produced abundant propanoic and butanoic acids, confirming that *Bifidobacteria* are effective SCFA producers (129, 130). These compounds increase membrane fluidity, causing leakage of intracellular contents and ultimately cell death (131). Propionic acid, in particular, generates reactive oxygen species (ROS), reduces ATP levels, and activates metacaspases, leading to mitochondrial-mediated apoptosis in fungal cells (132). Notably, propionic acid has been identified as a natural constituent of honey, contributing to its flavor and preservation (133).

Esters such as ethyl and propyl propionate have demonstrated antifungal activity (84), while other VOCs, like dimethyl disulfide and limonene, were also detected. Limonene damages fungal hyphae, causing cytoplasmic granulation, membrane detachment, and vacuole formation, ultimately leading to cell death (134, 135). Dimethyl disulfide exhibits antifungal activity by damaging membranes and inhibiting spore germination and hyphal growth (136, 137). *B. asteroides* 3CP-2B also produces methylpyrazines, aromatic hydrocarbons commonly found in foods and considered safe (138, 139). Pyrazine derivatives have broad biological activity, including antifungal effects (138, 140, 141). Gong et al. (142) demonstrated that methylpyrazine and dimethyl disulfide significantly inhibit fungal growth and spore germination. Transcriptome analysis showed that these VOCs downregulate ribosomal synthesis genes, activate the proteasome system, and suppress genes related to spore development, membrane synthesis, mitochondrial function, and toxin production. Exploring natural antifungal strategies may offer sustainable options for improving bee health. Microbial VOCs can be delivered using formulations designed to overcome their volatility and short lifespan. Recent studies have investigated the use of hydrogels and sprays containing microbial VOCs to control plant pathogenic fungi (123, 143). Similarly, antifungal hydrogel or spray formulations based on symbiotic bacteria like *B. asteroides* could represent a promising, eco-friendly strategy to manage fungal diseases such as Chalkbrood in honey bee colonies.

## 5 Conclusions

This study contributes to our understanding of the intricate relationship between honey bees and their gut microbiota. Through a preliminary characterization of *Bifidobacterium* strains isolated from the honey bee gut, we have demonstrated that certain isolates possess enzymatic activities involved in the detoxification of xenobiotics through hydrolysis or breakdown of harmful compounds. Additionally, several strains exhibited enzymatic capabilities that enhance nutrient bioavailability and facilitate the metabolism of specific sugars, such as mannose, lactose, raffinose, and xylose, that can otherwise be toxic to bees. Notably, *B. asteroides* 3CP-2B exhibited strong antifungal activity, suggesting its potential application as a probiotic supplement in honey bee diets or as an environmentally friendly biocontrol agent to reduce the incidence of fungal diseases such as chalkbrood. These findings lay a solid foundation for future biocontrol strategies based on honey bee-associated symbionts. However, further studies are essential to evaluate the safety of the VOCs produced by *B. asteroides* 3CP-2B, particularly their effects on healthy brood development. This will be crucial for developing safe and effective application strategies that do not disrupt the hive’s environmental balance or compromise colony productivity.

## Data Availability

The datasets presented in this study can be found in online repositories. The names of the repository/repositories and accession number(s) can be found below: https://www.ncbi.nlm.nih.gov/genbank/, PV053127 https://www.ncbi.nlm.nih.gov/genbank/, PV053134 https://www.ncbi.nlm.nih.gov/genbank/, PV053124 https://www.ncbi.nlm.nih.gov/genbank/, PV053131 https://www.ncbi.nlm.nih.gov/genbank/, PV053133 https://www.ncbi.nlm.nih.gov/genbank/, PV053126 https://www.ncbi.nlm.nih.gov/genbank/, PV053144 https://www.ncbi.nlm.nih.gov/genbank/, PV053125 https://www.ncbi.nlm.nih.gov/genbank/, PV053128 https://www.ncbi.nlm.nih.gov/genbank/, From PV056024 to PV056038.

## References

[1] Engel P Kwong WK McFrederick Q Anderson KE Barribeau SM Chandler JA. The bee microbiome: impact on bee health and model for evolution and ecology of host-microbe interactions. mBio. (2016) 7:10.1128/mbio.02164–15. doi: 10.1128/mbio.02164-15, PMID: 27118586 PMC4850275

[2] Romero S Nastasa A Chapman A Kwong WK Foster LJ. The honey bee gut microbiota: strategies for study and characterization. Insect Mol Biol. (2019) 28:455–72. doi: 10.1111/imb.12567, PMID: 30652367

[3] Motta EV Moran NA. The honeybee microbiota and its impact on health and disease. Nat Rev Microbiol. (2024) 22:122–37. doi: 10.1038/s41579-023-00990-3, PMID: 38049554 PMC10998682

[4] Kwong WK Moran NA. Gut microbial communities of social bees. Nat Rev Microbiol. (2016) 14:374–84. doi: 10.1038/nrmicro.2016.43, PMID: 27140688 PMC5648345

[5] Liberti J Kay T Quinn A Kesner L Frank ET Cabirol A. The gut microbiota affects the social network of honeybees. Nat Ecol Evol. (2022) 6:1471–9. doi: 10.1038/s41559-022-01840-w, PMID: 35995848 PMC7613669

[6] Ventura M Canchaya C Tauch A Chandra G Fitzgerald GF Chater KF. Genomics of Actinobacteria: tracing the evolutionary history of an ancient phylum. Microbiol Mol Biol Rev. (2007) 71:495–548. doi: 10.1128/MMBR.00005-07, PMID: 17804669 PMC2168647

[7] Endo A Futagawa-Endo Y Dicks LMT. Diversity of *Lactobacillus* and *Bifidobacterium* in feces of herbivores, omnivores and carnivores. Anaerobe. (2010) 16:590–6. doi: 10.1016/j.anaerobe.2010.10.005, PMID: 21034840

[8] Kopečný J Mrázek J Killer J. The presence of bifidobacteria in social insects, fish and reptiles. Folia Microbiol. (2010) 55:336–9. doi: 10.1007/s12223-010-0053-2, PMID: 20680566

[9] Bunesova V Vlkova E Rada V Killer J Musilova S. Bifidobacteria from the gastrointestinal tract of animals: differences and similarities. Benef Microbes. (2014) 5:377–88. doi: 10.3920/BM2013.0081, PMID: 24889892

[10] Alessandri G van Sinderen D Ventura M. The genus bifidobacterium: From genomics to functionality of an important component of the mammalian gut microbiota running title: Bifidobacterial adaptation to and interaction with the host. Comput Struct Biotechnol J. (2021) 19:1472–87. doi: 10.1016/j.csbj.2021.03.006, PMID: 33777340 PMC7979991

[11] Baffoni L Gaggìa F Alberoni D Cabbri R Nanetti A Biavati B. Effect of dietary supplementation of Bifidobacterium and Lactobacillus strains in Apis mellifera L. against Nosema ceranae. Benef Microbes. (2016) 7:45–51. doi: 10.3920/BM2015.0085, PMID: 26565084

[12] Chen J Wang J Zheng H. Characterization of *Bifidobacterium apousia* sp. nov., *Bifidobacterium choladohabitans* sp. nov., and *Bifidobacterium polysaccharolyticum* sp. nov., three novel species of the genus *Bifidobacterium* from honey bee gut. Systematic Appl Microbiol. (2021) 44:126247. doi: 10.1016/j.syapm.2021.126247, PMID: 34482030

[13] Scardovi V Trovatelli LD. New species of bifid bacteria from Apis mellifica L. and Apis indica F. A contribution to the taxonomy and biochemistry of the genus Bifidobacterium. Zentralbl Bakteriol Parasitenkd Infektionskr Hyg. (1969) 123:64–88., PMID: 4908741

[14] Modesto M Scarafile D Vásquez A Pukall R Neumann-Schaal M Pascarelli S. Phylogenetic characterization of Bifidobacterium kimbladii sp. nov., a novel species from the honey stomach of the honeybee Apis mellifera. Syst Appl Microbiol. (2025) 48:126579. doi: 10.1016/j.syapm.2025.126579, PMID: 39764984

[15] Wang T-Y Wang H Gu CT. Bifidobacterium apicola sp. nov., isolated from the gut of honeybee (Apis mellifera). Int J Syst Evol Microbiol. (2024) 74(12). doi: 10.1099/ijsem.0.006599, PMID: 39630494

[16] Jiang C-S Li CY Gu CT. Bifidobacterium apis sp. nov., isolated from the gut of honeybee (Apis mellifera). Int J Syst Evol Microbiol. (2024) 74(4). doi: 10.1099/ijsem.0.006358, PMID: 38661726

[17] Olofsson TC Modesto M Pascarelli S Scarafile D Mattarelli P Vasquez A. Bifidobacterium mellis sp. nov., isolated from the honey stomach of the honey bee Apis mellifera. Int J Systematic Evolutionary Microbiol. (2023) 73:5766. doi: 10.1099/ijsem.0.005766, PMID: 36884368

[18] Li TT Zhang HX Gu CT. Bifidobacterium mizhiense sp. nov., isolated from the gut of honeybee (Apis mellifera). Int J Syst Evol Microbiol. (2022) 72(5). doi: 10.1099/ijsem.0.005390, PMID: 35608970

[19] Killer J Kopečný J Mrázek J Koppová I Havlík J Benada O. Bifidobacterium actinocoloniiforme sp. nov. and Bifidobacterium bohemicum sp. nov., from the bumblebee digestive tract. Int J Syst Evol Microbiol. (2011) 61:1315–21. doi: 10.1099/ijs.0.022525-0, PMID: 20656822

[20] Killer J Kopecný J Mrázek J Rada V Benada O Koppová I. Bifidobacterium bombi sp. nov., from the bumblebee digestive tract. Int J Syst Evol Microbiol. (2009) 59:2020–4. doi: 10.1099/ijs.0.002915-0, PMID: 19567560

[21] Praet J Meeus I Cnockaert M Aerts M Smagghe G Vandamme P. Bifidobacterium commune sp. nov. isolated from the bumble bee gut. Antonie van Leeuwenhoek. (2015) 107:1307–13. doi: 10.1007/s10482-015-0425-3, PMID: 25753540

[22] Alberoni D Gaggìa F Baffoni L Modesto MM Biavati B Di Gioia D. Bifidobacterium xylocopae sp. nov. and Bifidobacterium aemilianum sp. nov., from the carpenter bee (Xylocopa violacea) digestive tract. Syst Appl Microbiol. (2019) 42:205–16. doi: 10.1016/j.syapm.2018.11.005, PMID: 30551956

[23] Li Y Song Q Yang H Wei Y Menghe B Liu W. Bifidobacterium favimelis sp. nov., isolated from black comb honey. Int J Syst Evol Microbiol. (2024) 74(11). doi: 10.1099/ijsem.0.006573, PMID: 39514412

[24] Mohr KI Tebbe CC. Diversity and phylotype consistency of bacteria in the guts of three bee species (Apoidea) at an oilseed rape field. Environ Microbiol. (2006) 8:258–72. doi: 10.1111/j.1462-2920.2005.00893.x, PMID: 16423014

[25] Anderson KE Johansson A Sheehan TH Mott BM Corby-Harris V Johnstone L. Draft genome sequences of two Bifidobacterium sp. from the honey bee (Apis mellifera). Gut Pathog. (2013) 5:42. doi: 10.1186/1757-4749-5-42, PMID: 24350840 PMC3878406

[26] Khan KA Al-Ghamdi AA Ghramh HA Ansari MJ Ali H Alamri SA. Structural diversity and functional variability of gut microbial communities associated with honey bees. Microbial Pathogenesis. (2020) 138:103793. doi: 10.1016/j.micpath.2019.103793, PMID: 31626917

[27] Lazarova S Lozanova L Neov B Shumkova R Balkanska R Palova N. Composition and diversity of bacterial communities associated with honey bee foragers from two contrasting environments. Bull Entomological Res. (2023) 113:693–702. doi: 10.1017/S0007485323000378, PMID: 37545319

[28] Alberoni D Baffoni L Gaggìa F Ryan PM Murphy K Ross PR. Impact of beneficial bacteria supplementation on the gut microbiota, colony development and productivity of Apis mellifera L. Benef Microbes. (2018) 9(2):269–78. doi: 10.3920/BM2017.0061, PMID: 29380644

[29] Bonilla-Rosso G Engel P. Functional roles and metabolic niches in the honey bee gut microbiota. Curr Opin Microbiol. (2018) 43:69–76. doi: 10.1016/j.mib.2017.12.009, PMID: 29309997

[30] Kešnerová L Mars RAT Ellegaard KM Troilo M Sauer U Engel P. Disentangling metabolic functions of bacteria in the honey bee gut. PloS Biol. (2017) 15:e2003467. doi: 10.1371/journal.pbio.2003467, PMID: 29232373 PMC5726620

[31] Zheng H Perreau J Powell JE Han B Zhang Z Kwong WK. Division of labor in honey bee gut microbiota for plant polysaccharide digestion. Proc Natl Acad Sci. (2019) 116:25909–16. doi: 10.1073/pnas.1916224116, PMID: 31776248 PMC6926048

[32] Anderson KE Ricigliano VA. Honey bee gut dysbiosis: a novel context of disease ecology. Curr Opin Insect Sci. (2017) 22:125–32. doi: 10.1016/j.cois.2017.05.020, PMID: 28805634

[33] Motta EVS Raymann K Moran NA. Glyphosate perturbs the gut microbiota of honey bees. Proc Natl Acad Sci U.S.A. (2018) 115:10305–10. doi: 10.1073/pnas.1803880115, PMID: 30249635 PMC6187125

[34] Kešnerová L Emery O Troilo M Liberti J Erkosar B Engel P. Gut microbiota structure differs between honeybees in winter and summer. ISME J. (2020) 14:801–14. doi: 10.1038/s41396-019-0568-8, PMID: 31836840 PMC7031341

[35] Baffoni L Alberoni D Gaggìa F Braglia C Stanton C Ross PR. Honeybee exposure to veterinary drugs: how is the gut microbiota affected? Microbiol Spectr. (2021) 9:e0017621. doi: 10.1128/Spectrum.00176-21, PMID: 34378962 PMC8552759

[36] Fernandez De Landa G Alberoni D Baffoni L Fernandez De Landa M Revainera PD Porrini LP. The gut microbiome of solitary bees is mainly affected by pathogen assemblage and partially by land use. Environ Microbiome. (2023) 18:38. doi: 10.1186/s40793-023-00494-w, PMID: 37098635 PMC10131457

[37] Hamdi C Balloi A Essanaa J Crotti E Gonella E Raddadi N. Gut microbiome dysbiosis and honeybee health. J Appl Entomology. (2011) 135:524–33. doi: 10.1111/j.1439-0418.2010.01609.x

[38] Castelli L Branchiccela B Garrido M Invernizzi C Porrini M Romero H. Impact of nutritional stress on honeybee gut microbiota, immunity, and nosema ceranae infection. Microb Ecol. (2020) 80:908–19. doi: 10.1007/s00248-020-01538-1, PMID: 32666305

[39] Daisley BA Chmiel JA Pitek AP Thompson GJ Reid G. Missing microbes in bees: how systematic depletion of key symbionts erodes immunity. Trends Microbiol. (2020) 28:1010–21. doi: 10.1016/j.tim.2020.06.006, PMID: 32680791

[40] Borum AE. Microbiota and its importance in honey bees. Bee Stud. (2021) 13:23–30. doi: 10.51458/BSTD.2021.14

[41] Maxfield-Taylor SA Mujic AB Rao S. First detection of the larval chalkbrood disease pathogen ascosphaera apis (Ascomycota: eurotiomycetes: ascosphaerales) in adult bumble bees. PloS One. (2015) 10:e0124868. doi: 10.1371/journal.pone.0124868, PMID: 25885679 PMC4401763

[42] Reynaldi FJ Lucia M Genchi Garcia ML. Ascosphaera apis, the entomopathogenic fungus affecting larvae of native bees (Xylocopa augusti): First report in South America. Rev Iberoam Micol. (2015) 32:261–4. doi: 10.1016/j.riam.2015.01.001, PMID: 25959551

[43] Chen D Guo R Xiong C Zheng Y Hou C Fu Z. Morphological and molecular identification of chalkbrood disease pathogen Ascosphaera apis in Apis cerana cerana. J Apicultural Res. (2018) 57:516–21. doi: 10.1080/00218839.2018.1475943

[44] Deneke Y. Review on chalkbrood disease of honey bee. Veterinary Med – Open J. (2023) 8:47–55. doi: 10.17140/VMOJ-8-176

[45] Tejerina MR Cabana MJ Benitez-Ahrendts MR. Strains of *Lactobacillus* spp. reduce chalkbrood in *Apis mellifera* . J Invertebrate Pathol. (2021) 178:107521. doi: 10.1016/j.jip.2020.107521, PMID: 33347864

[46] Sevim A Akpınar R Karaoğlu Ş.A Bozdeveci A Sevim E. Prevalence and phylogenetic analysis of Ascosphaera apis (Maassen ex Claussen) LS Olive & Spiltoi) isolates from honeybee colonies in Turkey. Biologia. (2022) 77:2689–99. doi: 10.1007/s11756-022-01114-7

[47] Das R Kumar R Kunal G Goldar S Dutta S Jha S. Detection of Ascosphaera apis, causing chalkbrood disease in the colonies of European honey bee, Apis mellifera in West Bengal, India. Sociobiology. (2023) 70:e9192–2. doi: 10.13102/sociobiology.v70i4.9129

[48] Karthik V Srinivasan MR Saminathan VR Karthikeyan S Balasubramani V. Morphological and Molecular Identification and Mating Type Detection of Chalkbrood Fungal Pathogen *Ascosphaera apis* in *Apis mellifera* L. @ in Southern India. Indian J Entomology. (2024) 87(2):349–55. doi: 10.55446/IJE.2024.2198

[49] Li Z Su S Hamilton M Yan L Chen Y. The ability to cause infection in a pathogenic fungus uncovers a new biological feature of honey bee viruses. J Invertebr Pathol. (2014) 120:18–22. doi: 10.1016/j.jip.2014.05.002, PMID: 24825460

[50] Cheng X Zhang L Luo J Yang S Deng Y Li J. Two pathogenic fungi isolated from chalkbrood samples and honey bee viruses they carried. Front Microbiol. (2022) 13:843842. doi: 10.3389/fmicb.2022.843842, PMID: 35495671 PMC9039454

[51] Kim DY Maeng S Cho S-J Park HJ Kim K Lee JK. The ascosphaera apis infection (Chalkbrood disease) alters the gut bacteriome composition of the honeybee. Pathogens. (2023) 12:734. doi: 10.3390/pathogens12050734, PMID: 37242403 PMC10224485

[52] Pavlović R Brodschneider R Goessler W Stanisavljević L Vujčić Z Zarić NM. Micronutrient deficiency may be associated with the onset of chalkbrood disease in honey bees. Insects. (2024) 15:269. doi: 10.3390/insects15040269, PMID: 38667399 PMC11050715

[53] Yoder JA Jajack AJ Cornacchione WS Dunn AL Cunningham EG Matchett CL. *In vitro* evaluation of sugar syrups, antibiotics, and miticides on growth of honey bee pathogen, Ascosphaera apis: Emphasis for chalkbrood prevention is on keeping bees healthy. Apidologie. (2014) 45:568–78. doi: 10.1007/s13592-014-0274-5

[54] Castagnino GLB Mateos A Meana A Montejo L Zamorano Iturralde LV Cutuli De Simón MT. Etiology, symptoms and prevention of chalkbrood disease: a literature review. Rev Bras saúde Prod anim. (2020) 21:e210332020. doi: 10.1590/S1519-9940210332020

[55] von Knoblauch T Jensen AB Mülling CKW Aupperle-Lellbach H Genersch E. Chalkbrood Disease Caused by Ascosphaera apis in Honey Bees (Apis mellifera)—Morphological and Histological Changes in Infected Larvae. Veterinary Sci. (2024) 11:415. doi: 10.3390/vetsci11090415, PMID: 39330794 PMC11436016

[56] Iorizzo M Lombardi SJ Ganassi S Testa B Ianiro M Letizia F. Antagonistic Activity against Ascosphaera apis and Functional Properties of Lactobacillus kunkeei Strains. Antibiotics (Basel). (2020) 9:262. doi: 10.3390/antibiotics9050262, PMID: 32443465 PMC7277644

[57] Ashraf SA Mahmood D Elkhalifa AEO Siddiqui AJ Khan MI Ashfaq F. Exposure to pesticide residues in honey and its potential cancer risk assessment. Food Chem Toxicol. (2023) 180:114014. doi: 10.1016/j.fct.2023.114014, PMID: 37659576

[58] Ansari MJ Al-Ghamdi A Usmani S Khan KA Alqarni AS Kaur M. *In vitro* evaluation of the effects of some plant essential oils on *Ascosphaera apis*, the causative agent of Chalkbrood disease. Saudi J Biol Sci. (2017) 24:1001–6. doi: 10.1016/j.sjbs.2016.04.016, PMID: 28663695 PMC5478295

[59] Khan SU Anjum SI Ansari MJ Khan MHU Kamal S Rahman K. Antimicrobial potentials of medicinal plant’s extract and their derived silver nanoparticles: A focus on honey bee pathogen. Saudi J Biol Sci. (2019) 26:1815–34. doi: 10.1016/j.sjbs.2018.02.010, PMID: 31762664 PMC6864162

[60] Pusceddu M Floris I Mangia NP Angioni A Satta A. *In Vitro* Activity of Several Essential Oils Extracted from Aromatic Plants against Ascosphaera apis. Veterinary Sci. (2021) 8:80. doi: 10.3390/vetsci8050080, PMID: 34068642 PMC8150519

[61] Krutmuang P Rajula J Pittarate S Chatima C Thungrabeab M Mekchay S. The inhibitory action of plant extracts on the mycelial growth of *Ascosphaera apis*, the causative agent of chalkbrood disease in Honey bee. Toxicol Rep. (2022) 9:713–9. doi: 10.1016/j.toxrep.2022.03.036, PMID: 35433272 PMC9006850

[62] Usta M. Biological control of honey bee diseases and pests. In: Melittology - new advances. IntechOpen (2023). doi: 10.5772/intechopen.1003750

[63] Boonmee T Sinpoo C Thayatham K Suanpoot P Disayathanoowat T Pettis JS. Atmospheric non-thermal plasma inactivation of Ascosphaera apis, the causative agent of chalkbrood disease in honeybee. Sci Rep. (2024) 14:1831. doi: 10.1038/s41598-024-52221-1, PMID: 38246935 PMC10800336

[64] Iorizzo M Letizia F Ganassi S Testa B Petrarca S Albanese G. Functional properties and antimicrobial activity from lactic acid bacteria as resources to improve the health and welfare of honey bees. Insects. (2022) 13:308. doi: 10.3390/insects13030308, PMID: 35323606 PMC8953987

[65] Abdi K Ben Said M Crotti E Masmoudi AS Cherif A. The promise of probiotics in honeybee health and disease management. Arch Microbiol. (2023) 205:73. doi: 10.1007/s00203-023-03416-z, PMID: 36705763

[66] Rodríguez MA Fernández LA Díaz ML Pérez M Corona M Reynaldi FJ. Microbiological and chemical characterization of water kefir: An innovative source of potential probiotics for bee nutrition. Rev Argent Microbiología. (2023) 55:176–80. doi: 10.1016/j.ram.2022.09.003, PMID: 36481105

[67] Iorizzo M Testa B Ganassi S Lombardi SJ Ianiro M Letizia F. Probiotic properties and potentiality of lactiplantibacillus plantarum strains for the biological control of chalkbrood disease. J Fungi (Basel). (2021) 7:379. doi: 10.3390/jof7050379, PMID: 34066127 PMC8151994

[68] Tejerina MR Cabana MJ Enríquez PA Benítez-Ahrendts MR Fonseca MI. Bacterial strains isolated from stingless bee workers inhibit the growth of apis mellifera pathogens. Curr Microbiol. (2024) 81:106. doi: 10.1007/s00284-024-03618-8, PMID: 38418777

[69] Iorizzo M Coppola F Pannella G Ganassi S Matarazzo C Albanese G. First Report on Antifungal Activity of Metschnikowia pulcherrima Against Ascosphaera apis, the Causative Agent of Chalkbrood Disease in Honeybee (Apis mellifera L.) Colonies. J Fungi. (2025) 11:336. doi: 10.3390/jof11050336, PMID: 40422670 PMC12112871

[70] Daisley BA Pitek AP Torres C Lowery R Adair BA Al KF. Delivery mechanism can enhance probiotic activity against honey bee pathogens. ISME J. (2023) 17:1382–95. doi: 10.1038/s41396-023-01422-z, PMID: 37311937 PMC10432525

[71] Pino A Benkaddour B Inturri R Amico P Vaccaro SC Russo N. Characterization of bifidobacterium asteroides isolates. Microorganisms. (2022) 10:655. doi: 10.3390/microorganisms10030655, PMID: 35336230 PMC8950671

[72] Dengiz B Killer J Havlík J Dobeš P Hyršl P. Selection of Probiotics for Honey Bees: The *In Vitro* Inhibition of Paenibacillus larvae, Melissococcus plutonius, and Serratia marcescens Strain Sicaria by Host-Specific Lactobacilli and Bifidobacteria. Microorganisms. (2025) 13:1159. doi: 10.3390/microorganisms13051159, PMID: 40431330 PMC12113734

[73] Moradi M Ownagh A. Antifungal Effects of Lactobacillus casei, Lactobacillus acidophilus and Bifidobacterium bifidum on the Ascospharea apis Causative Agent of Honey bee Chalkbrood Disease. J Veterinary Res. (2019) 74:273–82. doi: 10.22059/jvr.2019.217394.2533

[74] Ptaszyńska AA Borsuk G Zdybicka-Barabas A Cytryńska M Małek W. Are commercial probiotics and prebiotics effective in the treatment and prevention of honeybee nosemosis C? Parasitol Res. (2016) 115:397–406. doi: 10.1007/s00436-015-4761-z, PMID: 26437644 PMC4700093

[75] Kwong WK Mancenido AL Moran NA. Immune system stimulation by the native gut microbiota of honey bees. R Soc Open Sci. (2017) 4:170003. doi: 10.1098/rsos.170003, PMID: 28386455 PMC5367273

[76] El Khoury S Rousseau A Lecoeur A Cheaib B Bouslama S Mercier P-L. Deleterious Interaction Between Honeybees (Apis mellifera) and its Microsporidian Intracellular Parasite Nosema ceranae Was Mitigated by Administrating Either Endogenous or Allochthonous Gut Microbiota Strains. Front Ecol Evol. (2018) 6:58. doi: 10.3389/fevo.2018.00058

[77] Todorov SD Alves MV Bueno GCA Alves VF Ivanova IV. Bee-associated beneficial microbes—Importance for bees and for humans. Insects. (2024) 15:430. doi: 10.3390/insects15060430, PMID: 38921144 PMC11204305

[78] Zhang Z Schwartz S Wagner L Miller W. A greedy algorithm for aligning DNA sequences. J Comput Biol. (2000) 7:203–14. doi: 10.1089/10665270050081478, PMID: 10890397

[79] Resource Coordinators NCBI. Database resources of the national center for biotechnology information. Nucleic Acids Res. (2018) 46:D8–D13. doi: 10.1093/nar/gkx1095, PMID: 29140470 PMC5753372

[80] Johnson JS Spakowicz DJ Hong B-Y Petersen LM Demkowicz P Chen L. Evaluation of 16S rRNA gene sequencing for species and strain-level microbiome analysis. Nat Commun. (2019) 10:5029. doi: 10.1038/s41467-019-13036-1, PMID: 31695033 PMC6834636

[81] Lonsway DR. Preparation of routine media and reagents used in antimicrobial susceptibility testing. ClinMicroNow. (2023), 1–25. doi: 10.1002/9781683670438.cmph0082

[82] Torracca B Pedonese F Turchi B Fratini F Roberta N. Qualitative and quantitative evaluation of biogenic amines *in vitro* production by bacteria isolated from ewes’ milk cheeses. Eur Food Res Technol. (2018) 244(10):721–8. doi: 10.1007/s00217-017-2992-1

[83] Paca C Matei IA Diaconeasa Z Rotaru A Erler S Dezmirean DS. Biologically active extracts from different medicinal plants tested as potential additives against bee pathogens. Antibiotics. (2021) 10:960. doi: 10.3390/antibiotics10080960, PMID: 34439010 PMC8388991

[84] Ruiz-Moyano S Hernández A Galvan AI Córdoba MG Casquete R Serradilla MJ. Selection and application of antifungal VOCs-producing yeasts as biocontrol agents of grey mould in fruits. Food Microbiol. (2020) 92:103556. doi: 10.1016/j.fm.2020.103556, PMID: 32950150

[85] Serradilla MJ Martín A Hernandez A López-Corrales M Lozano M Córdoba M. Effect of the commercial ripening stage and postharvest storage on microbial and aroma changes of ‘Ambrunés’ Sweet cherries. J Agric Food Chem. (2010) 58:9157–63. doi: 10.1021/jf102004v, PMID: 23654239

[86] Bottacini F Milani C Turroni F Sánchez B Foroni E Duranti S. Bifidobacterium asteroides PRL2011 genome analysis reveals clues for colonization of the insect gut. PloS One. (2012) 7:e44229. doi: 10.1371/journal.pone.0044229, PMID: 23028506 PMC3447821

[87] Powell JE Martinson VG Urban-Mead K Moran NA. Routes of acquisition of the gut microbiota of the honey bee apis mellifera. Appl Environ Microbiol. (2014) 80:7378–87. doi: 10.1128/AEM.01861-14, PMID: 25239900 PMC4249178

[88] Raymann K Moran NA. The role of the gut microbiome in health and disease of adult honey bee workers. Curr Opin Insect Sci. (2018) 26:97–104. doi: 10.1016/j.cois.2018.02.012, PMID: 29764668 PMC6010230

[89] Devonshire AL Moores GD Ffrench-Constant RH. Detection of insecticide resistance by immunological estimation of carboxylesterase activity in Myzus persicae (Sulzer) and cross reaction of the antiserum with Phorodon humuli (Schrank) (Hemiptera: Aphididae). Bull Entomological Res. (1986) 76:97–107. doi: 10.1017/S0007485300015327

[90] Kishino S Takeuchi M Park S-B Hirata A Kitamura N Kunisawa J. Polyunsaturated fatty acid saturation by gut lactic acid bacteria affecting host lipid composition. Proc Natl Acad Sci U.S.A. (2013) 110:17808–13. doi: 10.1073/pnas.1312937110, PMID: 24127592 PMC3816446

[91] Milone J Rinkevich F Mcafee A Foster L Tarpy D. Differences in larval pesticide tolerance and esterase activity across honey bee (Apis mellifera) stocks. Ecotoxicology Environ Saf. (2020) 206:111213. doi: 10.1016/j.ecoenv.2020.111213, PMID: 32890926

[92] Bosch-Serra D Rodríguez MA Avilla J Sarasúa MJ Miarnau X. Esterase, glutathione S-transferase and NADPH-cytochrome P450 reductase activity evaluation in(Hemiptera: psyllidae) individual adults. Insects. (2021) 12:329. doi: 10.3390/insects12040329, PMID: 33917008 PMC8067761

[93] Claudianos C Ranson H Johnson RM Biswas S Schuler MA Berenbaum MR. A deficit of detoxification enzymes: pesticide sensitivity and environmental response in the honeybee. Insect Mol Biol. (2006) 15:615–36. doi: 10.1111/j.1365-2583.2006.00672.x, PMID: 17069637 PMC1761136

[94] Berenbaum MR Johnson RM. Xenobiotic detoxification pathways in honey bees. Curr Opin Insect Sci. (2015) 10:51–8. doi: 10.1016/j.cois.2015.03.005, PMID: 29588014

[95] Rand EE Smit S Beukes M Apostolides Z Pirk CWW Nicolson SW. Detoxification mechanisms of honey bees (Apis mellifera) resulting in tolerance of dietary nicotine. Sci Rep. (2015) 5:11779. doi: 10.1038/srep11779, PMID: 26134631 PMC4488760

[96] Gong Y Diao Q. Current knowledge of detoxification mechanisms of xenobiotic in honey bees. Ecotoxicology. (2017) 26:1–12. doi: 10.1007/s10646-016-1742-7, PMID: 27819118

[97] Chmiel JA Daisley BA Pitek AP Thompson GJ Reid G. Understanding the effects of sublethal pesticide exposure on honey bees: A role for probiotics as mediators of environmental stress. Front Ecol Evol. (2020) 8:22. doi: 10.3389/fevo.2020.00022

[98] Shamjana U Vasu DA Hembrom PS Nayak K Grace T. The role of insect gut microbiota in host fitness, detoxification and nutrient supplementation. Antonie van Leeuwenhoek. (2024) 117:71. doi: 10.1007/s10482-024-01970-0, PMID: 38668783

[99] Cui S Gu Z Wang W Tang X Zhang Q Mao B. Characterization of peptides available to different bifidobacteria. LWT. (2022) 169:113958. doi: 10.1016/j.lwt.2022.113958

[100] Li Y Leonard SP Powell JE Moran NA. Species divergence in gut-restricted bacteria of social bees. Proc Natl Acad Sci. (2022) 119:e2115013119. doi: 10.1073/pnas.2115013119, PMID: 35467987 PMC9170019

[101] Zheng H Nishida A Kwong WK Koch H Engel P Steele MI. Metabolism of toxic sugars by strains of the bee gut symbiont gilliamella apicola. mBio. (2016) 7:10.1128/mbio.01326–16. doi: 10.1128/mbio.01326-16, PMID: 27803186 PMC5090037

[102] Kubota M Tsuji M Nishimoto M Wongchawalit J Okuyama M Mori H. Localization of alpha-glucosidases I, II, and III in organs of European honeybees, Apis mellifera L., and the origin of alpha-glucosidase in honey. Biosci Biotechnol Biochem. (2004) 68:2346–52. doi: 10.1271/bbb.68.2346, PMID: 15564675

[103] Pokusaeva K O’Connell-Motherway M Zomer A Fitzgerald GF van Sinderen D. Characterization of two novel α-glucosidases from bifidobacterium breve UCC2003. Appl Environ Microbiol. (2009) 75:1135–43. doi: 10.1128/AEM.02391-08, PMID: 19114534 PMC2643558

[104] Stanley D Rejzek M Naested H Smedley M Otero S Fahy B. The role of alpha-glucosidase in germinating barley grains. Plant Physiol. (2011) 155:932–43. doi: 10.1104/pp.110.168328, PMID: 21098673 PMC3032477

[105] Guzik J Nakonieczny M Tarnawska M Bereś PK Drzewiecki S Migula P. The Glycolytic Enzymes Activity in the Midgut of Diabrotica virgifera virgifera (Coleoptera: Chrysomelidae) adult and their Seasonal Changes. J Insect Sci. (2015) 15:56. doi: 10.1093/jisesa/iev036

[106] Lezyk M Jers C Kjaerulff L Gotfredsen CH Mikkelsen MD Mikkelsen JD. Novel α-L-fucosidases from a soil metagenome for production of fucosylated human milk oligosaccharides. PloS One. (2016) 11:e0147438. doi: 10.1371/journal.pone.0147438, PMID: 26800369 PMC4723247

[107] Gama M do VF Alexandre Y Do N Pereira da Silva JM Castro DP. Digestive α-L-fucosidase activity in Rhodnius prolixus after blood feeding: effect of secretagogue and nutritional stimuli. Front Physiol. (2023) 14:1123414. doi: 10.3389/fphys.2023.1123414, PMID: 37538373 PMC10394381

[108] Grossmann GA Terra WR. α-Galactosidases from the larval midgut of *Tenebrio molitor* (Coleoptera) and *Spodoptera frugiperda* (Lepidoptera). Comp Biochem Physiol Part B: Biochem Mol Biol. (2001) 128:109–22. doi: 10.1016/S1096-4959(00)00306-7, PMID: 11163310

[109] Becker DJ Lowe JB. Fucose: biosynthesis and biological function in mammals. Glycobiology. (2003) 13:41R–53R. doi: 10.1093/glycob/cwg054, PMID: 12651883

[110] Pickard JM Chervonsky AV. Intestinal fucose as a mediator of host–microbe symbiosis. J Immunol. (2015) 194:5588–93. doi: 10.4049/jimmunol.1500395, PMID: 26048966 PMC4536407

[111] Hlalukana N Magengelele M Malgas S Pletschke BI. Enzymatic conversion of mannan-rich plant waste biomass into prebiotic mannooligosaccharides. Foods. (2021) 10:2010. doi: 10.3390/foods10092010, PMID: 34574120 PMC8468410

[112] Ricigliano VA Fitz W Copeland DC Mott BM Maes P Floyd AS. The impact of pollen consumption on honey bee (Apis mellifera) digestive physiology and carbohydrate metabolism. Arch Insect Biochem Physiol. (2017) 96:e21406. doi: 10.1002/arch.21406, PMID: 28833462

[113] Iorizzo M Pannella G Lombardi SJ Ganassi S Testa B Succi M. Inter- and Intra-Species Diversity of Lactic Acid Bacteria in Apis mellifera ligustica Colonies. Microorganisms. (2020) 8:1578. doi: 10.3390/microorganisms8101578, PMID: 33066358 PMC7602248

[114] Iorizzo M Testa B Lombardi SJ Ganassi S Ianiro M Letizia F. Antimicrobial activity against Paenibacillus larvae and functional properties of Lactiplantibacillus plantarum strains: Potential benefits for honeybee health. Antibiotics. (2020) 9:442. doi: 10.3390/antibiotics9080442, PMID: 32722196 PMC7460353

[115] Tan K Guo YH Nicolson SW Radloff SE Song QS Hepburn HR. Honeybee (Apis cerana) foraging responses to the toxic honey of Tripterygium hypoglaucum (Celastraceae): changing threshold of nectar acceptability. J Chem Ecol. (2007) 33:2209–17. doi: 10.1007/s10886-007-9384-0, PMID: 18058178

[116] Taylor MA Robertson AW Biggs PJ Richards KK Jones DF Parkar SG. The effect of carbohydrate sources: Sucrose, invert sugar and components of mānuka honey, on core bacteria in the digestive tract of adult honey bees (Apis mellifera). PloS One. (2019) 14:e0225845. doi: 10.1371/journal.pone.0225845, PMID: 31800608 PMC6892475

[117] Lee FJ Rusch DB Stewart FJ Mattila HR Newton ILG. Saccharide breakdown and fermentation by the honey bee gut microbiome. Environ Microbiol. (2015) 17:796–815. doi: 10.1111/1462-2920.12526, PMID: 24905222

[118] Hidalgo-Cantabrana C Delgado S Ruiz L Ruas-Madiedo P Sánchez B Margolles A. Bifidobacteria and their health-promoting effects. Microbiol Spectr. (2017) 5(3). doi: 10.1128/microbiolspec.BAD-0010-2016, PMID: 28643627 PMC11687494

[119] Herranz YS Amore M Del CC. Compounds with antifungal activity, which are produced by bifidobacterium spp (2006). Available online at: https://patents.google.com/patent/WO2006070041A2/en (Accessed July 3, 2025).

[120] Qiao N Yu L Zhang C Wei C Zhao J Zhang H. A comparison of the inhibitory activities of Lactobacillus and Bifidobacterium against Penicillium expansum and an analysis of potential antifungal metabolites. FEMS Microbiol Lett. (2020) 367:fnaa130. doi: 10.1093/femsle/fnaa130, PMID: 32845333

[121] Yu L Qiao N Wei C Hu Q Zhai Q Yan B. Underlying mechanisms of the antagonistic effects of *Bifidobacterium adolescentis* CCFM1108 on *Penicillium expansum*: Based on comparative transcriptome analysis. Food Bioscience. (2022) 47:101693. doi: 10.1016/j.fbio.2022.101693

[122] Kim H Maigoro AY Lee J-H Frunze O Kwon H-W. The improving effects of probiotic-added pollen substitute diets on the gut microbiota and individual health of honey bee (Apis mellifera L.). Microorganisms. (2024) 12:1567. doi: 10.3390/microorganisms12081567, PMID: 39203409 PMC11356693

[123] Zhao X Zhou J Tian R Liu Y. Microbial volatile organic compounds: Antifungal mechanisms, applications, and challenges. Front Microbiol. (2022) 13:922450. doi: 10.3389/fmicb.2022.922450, PMID: 35910607 PMC9337857

[124] Pokusaeva K Fitzgerald GF van Sinderen D. Carbohydrate metabolism in bifidobacteria. Genes Nutr. (2011) 6:285–306. doi: 10.1007/s12263-010-0206-6, PMID: 21484167 PMC3145055

[125] Rane HS Bernardo SM Walraven CJ Lee SA. *In Vitro* Analyses of Ethanol Activity against Candida albicans Biofilms. Antimicrob Agents Chemother. (2012) 56:4487–9. doi: 10.1128/AAC.00263-12, PMID: 22615286 PMC3421629

[126] Zinn M-K Bockmühl D. Did granny know best? Evaluating the antibacterial, antifungal and antiviral efficacy of acetic acid for home care procedures. BMC Microbiol. (2020) 20:265. doi: 10.1186/s12866-020-01948-8, PMID: 32847510 PMC7447605

[127] Suwannarach N Kumla J Bussaban B Nuangmek W Matsui K Lumyong S. Biofumigation with the endophytic fungus *Nodulisporium* spp. CMU-UPE34 to control postharvest decay of citrus fruit. Crop Prot. (2013) 45:63–70. doi: 10.1016/j.cropro.2012.11.015

[128] Williamson DA Carter GP Howden BP. Current and emerging topical antibacterials and antiseptics: agents, action, and resistance patterns. Clin Microbiol Rev. (2017) 30:827–60. doi: 10.1128/CMR.00112-16, PMID: 28592405 PMC5475228

[129] Usta-Gorgun B Yilmaz-Ersan L. Short-chain fatty acids production by *Bifidobacterium* sp*ecies* in the presence of salep. Electronic J Biotechnol. (2020) 47:29–35. doi: 10.1016/j.ejbt.2020.06.004

[130] Fusco W Lorenzo MB Cintoni M Porcari S Rinninella E Kaitsas F. Short-chain fatty-acid-producing bacteria: key components of the human gut microbiota. Nutrients. (2023) 15:2211. doi: 10.3390/nu15092211, PMID: 37432351 PMC10180739

[131] Bhattacharyya A Sinha M Singh H Patel RS Ghosh S Sardana K. Mechanistic insight into the antifungal effects of a fatty acid derivative against drug-resistant fungal infections. Front Microbiol. (2020) 11:2116. doi: 10.3389/fmicb.2020.02116, PMID: 33013771 PMC7505954

[132] Yun J Lee DG. A novel fungal killing mechanism of propionic acid. FEMS Yeast Res. (2016) 16:fow089. doi: 10.1093/femsyr/fow089, PMID: 27707757

[133] Costa Dos Santos A Carina Biluca F Brugnerotto P Valdemiro Gonzaga L Carolina Oliveira Costa A Fett R. Brazilian stingless bee honey: Physicochemical properties and aliphatic organic acids content. Food Res Int. (2022) 158:111516. doi: 10.1016/j.foodres.2022.111516, PMID: 35840224

[134] Chee HY Kim H Lee MH. *In vitro* Antifungal Activity of Limonene against Trichophyton rubrum. Mycobiology. (2009) 37:243–6. doi: 10.4489/MYCO.2009.37.3.243, PMID: 23983542 PMC3749397

[135] Giorgio A De Stradis A Lo Cantore P Iacobellis NS. Biocide effects of volatile organic compounds produced by potential biocontrol rhizobacteria on Sclerotinia sclerotiorum. Front Microbiol. (2015) 6:1056. doi: 10.3389/fmicb.2015.01056, PMID: 26500617 PMC4594563

[136] Tyagi S Lee K-J Shukla P Chae J-C. Author Correction: Dimethyl disulfide exerts antifungal activity against Sclerotinia minor by damaging its membrane and induces systemic resistance in host plants. Sci Rep. (2020) 10:18183. doi: 10.1038/s41598-020-74901-4, PMID: 33082464 PMC7576206

[137] Chen X Liu J Chen AJ Wang L Jiang X Gong A. Burkholderia ambifaria H8 as an effective biocontrol strain against maize stalk rot via producing volatile dimethyl disulfide. Pest Manage Sci. (2024) 80:4125–36. doi: 10.1002/ps.8119, PMID: 38578571

[138] Adams TB Doull J Feron VJ Goodman JI Marnett LJ Munro IC. The FEMA GRAS assessment of pyrazine derivatives used as flavor ingredients. Flavor and Extract Manufacturers Association. Food Chem Toxicol. (2002) 40:429–51. doi: 10.1016/s0278-6915(01)00123-5, PMID: 11893403

[139] Dolezal M Zitko J. Pyrazine derivatives: a patent review (June 2012 - present). Expert Opin Ther Pat. (2015) 25:33–47. doi: 10.1517/13543776.2014.982533, PMID: 25523365

[140] Kucerova-Chlupacova M Kunes J Buchta V Vejsova M Opletalova V. Novel pyrazine analogs of chalcones: synthesis and evaluation of their antifungal and antimycobacterial activity. Molecules. (2015) 20:1104–17. doi: 10.3390/molecules20011104, PMID: 25587786 PMC6272410

[141] Su F Su Z Zhao Q Zhao Z Wu Z Zhao M. Synthesis, thermal property and antifungal evaluation of pyrazine esters. Arabian J Chem. (2022) 15:104351. doi: 10.1016/j.arabjc.2022.104351

[142] Gong Y Liu J-Q Xu M-J Zhang C-M Gao J Li C-G. Antifungal volatile organic compounds from streptomyces setonii WY228 control black spot disease of sweet potato. Appl Environ Microbiol. (2022) 88:e0:231721. doi: 10.1128/aem.02317-21, PMID: 35108080 PMC8939359

[143] Parafati L Cirvilleri G Restuccia C Wisniewski M. Potential role of exoglucanase genes (WaEXG1 and waEXG2) in the biocontrol activity of wickerhamomyces anomalus. Microb Ecol. (2017) 73:876–84. doi: 10.1007/s00248-016-0887-5, PMID: 27816988

