# Supplementary material for: Ascosphaera apis as a target for the antifungal activity of symbiotic Bifidobacteria in honey bees
Source: Front Insect Sci. 2025 Oct 1;5:1669013. doi: 10.3389/finsc.2025.1669013 (PMC12521233; doi:10.3389/finsc.2025.1669013)
Supplement: Supplementary file 1 [file Table1.docx]

Supplementary Material

**Supplementary Table 1.** Volatile organic compounds (VOCs) produced by *Bifidobacterium asteroides* 3CP-2B and detected by HS-SPME/GC-MS after 5 days of incubation.

| **Volatile compounds** | **Code** | **%RPA** | **^a^KIsp/KIt** | **^b^ID** |
| --- | --- | --- | --- | --- |
| **Aldehydes** |  |  |  |  |
| 2-Methyl-2-Butenal | Ald1 | 0.1 | 1115/1116 | RI/MS |
| Benzaldehyde | Ald2 | 0.2 | 1518/1520 | RI/MS/S |
| **Ketones** |  |  |  |  |
| Acetone | K1 | 0.1 | 812/812 | RI/MS/S |
| 2-Heptanone | K2 | 0.1 | 1187/1187 | RI/MS/S |
| 2-Nonanone | K3 | 0.1 | 1839/1835 | RI/MS/S |
| **Esters** |  |  |  |  |
| Ethyl acetate | E1 | 0.4 | 860/863 | RI/MS/S |
| Ethyl propionate | E2 | 3.1 | 942/946 | RI/MS/S |
| Propyl propionate | E3 | 0.7 | 1045/1047 | RI/MS |
| Isobutyl propionate | E4 | 0.1 | 1082/1087 | RI/MS |
| Isoamyl acetate | E5 | 0.1 | 1124/1127 | RI/MS/S |
| Butyl propionate | E6 | 0.1 | 1129/1132 | RI/MS |
| Isoamyl propionate | E7 | 0.1 | 1190/1192 | RI/MS/S |
| **Alcohols** |  |  |  |  |
| Ethanol | Alc1 | 25.0 | 933/934 | RI/MS/S |
| 2-Butanol | Alc2 | 0.1 | 1018/1020 | RI/MS/S |
| 1-Propanol | Alc3 | 2.3 | 1033/1037 | RI/MS/S |
| Isobutyl alcohol | Alc4 | 0.1 | 1106/1107 | RI/MS/S |
| 1-Butanol | Alc5 | 0.7 | 1142/1142 | RI/MS/S |
| Isoamyl alcohol | Alc6 | 1.7 | 1212/1215 | RI/MS/S |
| 2-Heptanol | Alc7 | 0.1 | 1318/1320 | RI/MS/S |
| 1-Hexanol | Alc8 | 0.1 | 1362/1360 | RI/MS/S |
| 2-Ethylhexanol | Alc9 | 0.1 | 1480/1484 | RI/MS/S |
| 1-Nonanol | Alc10 | 0.1 | 1645/1644 | RI/MS/S |
| Benzyl Alcohol | Alc11 | 0.1 | 1838/1837 | RI/MS/S |
| Phenethyl alcohol | Alc12 | 0.1 | 1916/1915 | RI/MS/S |
| **Acids** |  |  |  |  |
| Acetic acid | A1 | 17.3 | 1448/1445 | RI/MS/S |
| Propanoic acid | A2 | 45.8 | 1530/1534 | RI/MS |
| 2-Methyl-propanoic acid | A3 | 0.2 | 1581/1581 | RI/MS/S |
| Butanoic acid | A4 | 0.3 | 1632/1630 | RI/MS/S |
| Pentanoic acid | A5 | 0.1 | 1721/1723 | RI/MS/S |
| Hexanoic acid | A6 | 0.1 | 1826/1824 | RI/MS/S |
| **Pyrazines** |  |  |  |  |
| Methylpyrazine | Pyr1 | 0.1 | 1253/1252 | RI/MS/S |
| 2,5-Dimethylpyrazine | Pyr2 | 0.1 | 1324/1324 | RI/MS/S |
| 2-Methyl-3-ethylpyrazine | Pyr3 | 0.1 | 1399/1397 | RI/MS |
| **Furans** |  |  |  |  |
| 2-Furanmethanol | F1 | 0.1 | 1665/1666 | RI/MS/S |
|  |  |  |  |  |
| **Sulfur compouds** |  |  |  |  |
| Dimethyldisulfide | S1 | 0.1 | 1074/1072 | RI/MS/S |
| 3-Methylthiophene | S2 | 0.1 | 1115/1115 | RI/MS |
| **Others** |  |  |  |  |
| Styrene | O2 | 0.1 | 1251/1250 | RI/MS/S |
| Limonene | O3 | 0.1 | 1200/1199 | RI/MS/S |

Mean values of 3 samples are calculated as RPA (%). ^a^RIsp: Relative retention indices calculated against n-alkanes (C_8_–C_20_) on HP-Innowax column; RIt: Relative retention indices on polar column reported in literature ^b^Identification method as indicated by the following: RI: Kovats retention index on a on HP-Innowax column; MS: NIST and Wiley libraries spectra; S: co-injection with authentic standard compounds, where commercially available, on the HP-Innowax column. For each metabolite the coefficient of variability of determinations, evaluated as relative standard deviation, was in all cases <10%.
